# MicroRNA‐590‐3p inhibits trophoblast‐dependent maternal spiral artery remodeling by repressing low‐density lipoprotein receptor‐related protein 6

**DOI:** 10.1002/mgg3.491

**Published:** 2018-11-08

**Authors:** Yinghong Zhang, Xianzhen Pan, Xiaoyan Yu, Lei Li, Hongmei Qu, Shuhong Li

**Affiliations:** ^1^ Department of Obstetrics The Affiliated Yantai Yuhuangding Hospital of Qingdao University Yantai China; ^2^ Department of Obstetrics Shandong Provincial Hospital Affiliated to Shandong University Jinan China

**Keywords:** low‐density lipoprotein receptor‐related protein 6, microRNA‐590‐3p, spiral artery remodeling, trophoblast

## Abstract

**Background:**

The remodeling of maternal spiral artery following embryo implantation, which relies on well‐regulated trophoblast functions, is a pivotal process to ensure a successful pregnancy. Low‐density lipoprotein receptor‐related protein 6 (LRP6) and microRNAs (miRNAs, miRs) are suggested to be involved in angiogenesis and several vascular diseases; however, their functions in the control of trophoblast remain elusive. We therefore aimed to examine the roles of LRP6 and miR‐590‐3p in the regulation of trophoblast during the remodeling of maternal spiral artery.

**Methods:**

HTR‐8/SVneo cell, a trophoblast cell line, was utilized to study the effects of LRP6 and miR‐590‐3p on apoptosis, cell proliferation, migration, invasion, as well as tube formation. Expression of angiogenic factors placental growth factor (PlGF), matrix metalloproteinases (MMPs), vascular endothelial growth factor (VEGF), and activities of canonical Wnt/β‐catenin signaling pathway, which were implicated in the process of artery remodeling, were also examined.

**Results:**

MiR‐590‐3p directly targeted 3′ untranslated region (3′‐UTR) of LRP6 mRNA and repressed LRP6 expression, which in turn inhibited proliferation, migration, invasion, as well as tube formation, and resulted in apoptosis in HTR‐8/SVneo cells. Further, inhibition of LRP6 through miR‐590‐3p significantly suppressed the expression of PlGF, MMPs, and VEGF and reduced the activation of Wnt/β‐catenin signaling pathway.

**Conclusion:**

MicroRNAs‐590‐3p may inhibit trophoblast‐dependent maternal spiral artery remodeling, via both trophoblast invasion and endovascular formation, by repressing LRP6.

## 1 INTRODUCTION

Proper remodeling of spiral artery during early pregnancy is crucial for successful fetal development and pregnancy outcomes. Following the implantation of embryo, villous cytotrophoblasts may come together to form syncytiotrophoblasts, or otherwise differentiate into extra villous trophoblast (EVT) cells which grow out of villi and further invade into maternal tissue. EVT cells can be separated into two subpopulations: (a) interstitial EVTs (inEVTs) that invade into maternal decidual stroma and some part of myometrium; (b) endovascular EVTs (enEVTs) that migrate to spiral arteries and switch to endothelial phenotype. Both subtypes have been implicated in the transformation of maternal arteries to vessels which are characterized by high perfusion with low resistance that ensures sufficient supply of blood and nutrient to the fetus (Pijnenborg, Vercruysse, & Hanssens, 2006). Deficit of this process is closely associated with pregnancy‐related disorders including preeclampsia, restriction of fetal growth, and early pregnancy loss (Kaufmann, Black, & Huppertz, 2003; Orozco et al., 2009; van den Brule et al., 2005). Hence, insights into the process of trophoblast invasion and vascular transformation are highly valuable for early diagnosis of or effective treatments for pregnancy‐associated complications.

The low‐density lipoprotein (LDL) receptor‐related protein 6 (LRP6) was cloned based on its homology with the LDL receptor (LDLR) (Brown et al., 1998). The LRP of family proteins is a group of cell surface LDLRs that have similar structures but exert diverse functions in development and disease mechanisms in various organs and cell types. Mutations in LRP6 protein have been shown to result in a number of cardiovascular diseases (Caira et al., 2006; Rajamannan, Subramaniam, Caira, Stock, & Spelsberg, 2005). LRP6 belongs to a family of LRPs, most of which are single‐span transmembrane proteins that participate in the canonical Wnt/β‐catenin signaling (He, Semenov, Tamai, & Zeng, 2004). Both LRP6 and Wnt signaling are key players in the vascular development (Gessert & Kuhl, 2010). It is recently discovered that Kremen 2 (KRM2) promotes LRP6‐mediated Wnt signaling and is necessary for the induction of neural crest induction in Xenopus (Hassler et al., 2007). Wnt signaling also contributes to the development of heart valve, induction of osteogenic gene at particular stages of valve development, and remodeling of semilunar valves and atrioventricular (Alfieri, Cheek, Chakraborty, & Yutzey, 2010). All of these developmental findings demonstrated the key role of this signaling pathway in the development of heart valve and cardiac systems. Studies have also shown that postnatal expression of LRP6 is normally low except in pathological conditions.

On the other hand, with regard to trophoblast development, the LRPs reportedly regulate the activity of cell surface plasminogen activator in human trophoblast cells (Zhang et al., 1998), where LRPs play critical roles in embryo implantation through internalization and degradation of urokinase (u‐PA) and plasminogen activator inhibitor type‐1 (PAI‐1) complexes (Herz, Clouthier, & Hammer, 1992). Further, activation of the canonical Wnt pathway was shown to facilitate invasive differentiation of human trophoblast cells (Pollheimer et al., 2006). However, to the best of our knowledge, the exact functions of LRP6 in the remodeling of spiral artery remain unknown. We thus aimed to examine the roles of LRP6 in trophoblast regulation during the remodeling of maternal spiral artery.

MicroRNAs (miRNAs, miRs) refer to a family of small non‐protein‐coding RNAs (∼22 nucleotides) majority of which are negative regulators of gene expression through direct binding to the 3′ untranslated regions (3′‐UTRs) of target mRNAs (Mohr & Mott, 2015). miRNAs are believed to exert critical functions in various biological processes, including many pregnancy‐related diseases (Chen & Wang, 2013). For instance, overexpression of trophoblast stem cell‐enriched miRNAs could promote trophoblast fate in embryonic stem cells (Nosi, Lanner, Huang, & Cox, 2017). In preeclampsia, upregulation of miR‐299 suppressed the invasion and migration of HTR‐8/SVneo trophoblast cells by targeting HDAC2 (Gao, She, Wang, Li, & Zhang, 2017). Moreover, miR‐30a‐3p was found to be overexpressed in the placentas of preeclampsia patients and affected trophoblast invasion and apoptosis targeting IGF‐1 (Niu et al., 2017). In the current study, we were also interested in the function of a novel miR‐590‐3p during maternal spiral artery remodeling.

## 2 MATERIALS AND METHODS

### 2.1 Ethical compliance

This study was approved by the ethics committee of the Affiliated Yantai Yuhuangding Hospital of Qingdao University.

### 2.2 Cell culture

The human first trimester EVT cell lines HTR‐8/SVneo and TEV1, widely used as in vitro model for trophoblast‐dependent maternal spiral artery remodeling (Telugu et al., 2013), were maintained in RPMI 1640 (Hyclone, USA) supplemented with 100 IU/ml penicillin, 100 mg/ml streptomycin, and 10% fetal bovine serum (FBS, Gibco‐BRL‐Life Technologies, USA) at 37°C in 5% CO_2_. Every 48 hr culture medium was replaced with fresh ones. MystiCq miR‐590‐3p assay kit (MIRAP00576; Sigma‐Aldrich, St. Louis, MO, USA) was used to measure expressions of miR‐590‐3p, normalized against RNU6 control microRNA assay (MIRCP00001; Sigma‐Aldrich, USA). The Mission lentiviral miR‐590‐3p mimic (HLMIR0815; Sigma‐Aldrich, USA) and negative control (NCLMIR001; Sigma‐Aldrich, USA) were packaged into lentivirus following manufacturer's instructions, which were then used to establish stable cell lines.

### 2.3 qRT‐PCR

Extraction of RNA, reverse transcription, real‐time quantitative‐PCR assay, and data analysis were conducted using previously described protocols (Zhao et al., 2014). The conditions for PCR were as follows: 95°C for 30 s, 40 cycles at 95°C for 30 s, 65°C for 30 s, and extension at 72°C for 60 s, using the StepOne real‐time PCR System. Relative gene expressions were analyzed based on the comparative 2^−△△Ct^ method using GAPDH as the internal control. Primer pairs used in the study were as follows: LRP6 sense 5′‐AGG CAC TTA CTT CCC TGC AA‐3′, antisense 5′‐GGG CAC AGG TTC TGA ATC AT‐3′; matrix metalloproteinase (MMP)‐2 sense 5′‐TGA TCT TGA CCA GAA TAC CAT CGA‐3′, antisense 5′‐GGC TTG CGA GGG AAG AAG TT‐3′; MMP‐9 sense 5′‐TTT GAG TCC GGT GGA CGA TG‐3′, antisense 5′‐GCT CCT CAA AGA CCG AGT CC‐3′; placental growth factor (PlGF) sense 5′‐GGG GAA GAG GAG GAG AGA GA‐3′, antisense 5′‐CTC TCA CGT TGT TGA AGG CA‐3′; vascular endothelial growth factor (VEGF) sense 5′‐GCC TCG GGC TTG TCA CAT TTT‐3′, antisense 5′‐CCC TGA TGA GAT CGA GTA CAT CT‐3′; β‐catenin sense 5′‐GTG CTA TCT GTC TGC TCT AGT‐3′, antisense 5′‐CTT CCT GTT TAG TTG CAG CAT‐3′; GAPDH sense 5′‐ACA AAC ATA AGC AAG GCA CAG‐3′, antisense 5′‐GGT CGG AGT CAA CGG ATT TG‐3′.

### 2.4 Western blot

Total proteins were purified from the cell cultures using RIPA. Concentration of total protein was determined by the BCA assay. Protein samples (50 mg each) were run on 12% sodium dodecyl sulfate‐polyacrylamide gel and then transferred onto nitrocellulose membrane, which were subsequently incubated with antibodies against LRP6 (1:200, sc‐25317), MMP‐2 (1:500, sc‐13594), MMP‐9 (1:1,000, sc‐12759), PlGF (1:200, sc‐1880), VEGF (1:500, sc‐152), β‐catenin (1:1,000, sc‐7963), or GADPH (1:500, sc‐47724), respectively, at room temperature for 1 hr. Incubated membranes were developed with appropriate horseradish peroxidase‐conjugated secondary antibody for 1 hr at room temperature. Final bands were visualized by enhanced chemiluminescence procedure. All antibodies were obtained from Santa Cruz Biotechnology (Santa Cruz, CA, USA).

### 2.5 Dual‐luciferase reporter assay

Wild‐type (LRP6‐wtUTR) and mutated (LRP6‐mutUTR) miR‐590‐3p targeting sequences from LRP6 3′‐UTR were cloned into the downstream of a luciferase reporter gene on the pGL3‐enhancer plasmid using the pMir‐Report vector kit (Applied Biosystems, Carlsbad, CA, USA). Cells at the density of 10^5^ per well were plated in 24‐well plate and transfected with indicated luciferase constructs using Lipofectamine 2000 (Invitrogen, Carlsbad, CA, USA), then allowed 24 hr to grow after the transfection before various experiments. The activities of luciferase were determined using a dual‐luciferase reporter assay kit (Promega, Madison, WI, USA) following the manufacturer's instructions.

### 2.6 Cell proliferation assay

Cell Counting Kit‐8 (CCK‐8; Dojindo, Japan) was used to assess cell proliferation capacity. Briefly, cells were seeded to 96‐well plates at the density of 5 × 10^4^ cells per well with 100 ml complete medium. After 24 hr, 100 ml CCK‐8 solution was added into each well, followed by incubation in 5% CO_2_ for another 2 hr at 37°C. Absorbance at 450 nm was measured with a microplate reader.

### 2.7 Apoptosis assay

Costaining with Hoechst33342 and propidium iodide (PI, Life Technologies) was employed to examine cell apoptosis. Glass coverslips seeded with cells were placed in 6‐well plates at a density of 1 × 10^5^ cells per well, followed by 30 min incubation in medium containing 1 μg/ml Hoechst33342 and 1 μg/ml PI, then briefly washed with PBS. Fluorescence was examined using a fluorescent microscope. The intensity of fluorescent signal was quantified from nine regions of interest randomly picked from three independent experiments.

### 2.8 Caspase‐3 colorimetric assay

Additional assessment of cell apoptosis was performed using Caspase‐3 colorimetric assay kit (KeyGEN BioTECH, Nanjing, China) following the provided protocol. In brief, total proteins (200 mg) from cells were diluted in 50 ml lysis buffer. Reaction Buffer (50 ml) and Caspase‐3 Substrate (5 ml) were then added to each sample sequentially. After 4 hr incubation in dark at 37°C, absorbance at 405 nm was measured with a microplate reader.

### 2.9 Cell migration assay

The ability of cell migration was evaluated using the wound healing assay. Briefly, a wound was generated by a linear cut through confluent cells using the tip of a 200 μl pipette which was defined as the cell boundary at time zero. After 24 hr, the migration into the scratches was measured as the distance between the front edges of cells under an inverted phase‐contrast microscope (Olympus, Japan).

### 2.10 Cell invasion assay

Resuspension of 2 × 10^5^ cells in serum‐free RPMI1640 (200 ml) was added to 24‐well transwell insert (pore size 8 mm, Corning, USA) with 600 ml complete culture medium in the lower compartment. Cells were maintained in a 5% CO_2_ humidified incubator at 37°C for 24 hr, followed by exposure to 5‐ethynyl‐2’‐deoxyuridine (EdU, 20 μM) for another 4 hr at 37°C. Inserts were then detached from the plate and visualized using the ENU kit (Invitrogen). For each well, cells were quantified from six randomly picked representative fields. Invasion rate was calculated and normalized to the appropriate control.

### 2.11 Tube formation assay

HTR‐8/SVneo is likely to show endothelial behavior such as tube‐like formation on Matrigel substance (Highet, Zhang, Heinemann, & Roberts, 2012). 96‐well plates coated with 100 ml Matrigel were rested at 37°C for 1 hr. A total of 1,104 transfected cells were seeded onto the upper layer of the formed gel. After 6 hr incubation at 37°C, plates were examined and photographed. The number of branching points giving rise to three or more tubules was counted.

### 2.12 Statistical analysis

Data were presented as mean + standard deviation (*SD*), and statistical analysis was conducted using GraphPad Prism 5. Differences between two groups were determined using Student *t* test or analysis of variance where appropriate (ANOVA). *p* values <0.05 were considered statistically significant.

## 3 RESULTS

### 3.1 MiR‐590‐3p inhibits LRP6 expression via its 3′‐untranslated region (UTR)

**Figure 1:**
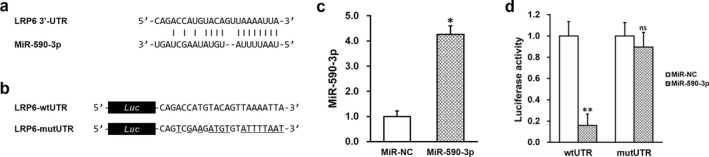
Lipoprotein receptor‐related protein 6 (LRP6) mRNA 3′‐untranslated region (UTR) is directly recognized by miR‐590‐3p. (a) Predicted miR‐590‐3p targeting sequence on the 3′‐UTR of LRP6 mRNA. (b) Wild‐type (wtUTR) targeting sequence of miR‐590‐3p on 3′‐UTR of LRP6 mRNA and the mutated version (mutUTR) were cloned at the downstream of the luciferase open reading frame (Luc). (c) Levels of miR‐590‐3p in HTR‐8/SVneo cells were examined after stable expression of negative control miR (miR‐NC) or miR‐590‐3p mimic, respectively. (d) Luciferase activities of LRP6‐wtUTR or LRP6‐mutUTR constructs in HTR‐8/SVneo cells were examined after stable expression of negative control miR (miR‐NC) or miR‐590‐3p mimic, respectively. Values are mean + *SD* from three independent experiments. **p < 0.01, *p < 0.05, ns not significant, compared to miR‐NC

We were interested in the role of LRP6 in spiral artery remodeling, and whether miRNAs were also involved in this process. We therefore examined the sequence of LRP6 mRNA, using the miRanda algorithm (Enright et al., 2003) to identify miRNAs that could potentially target LRP6 mRNA. MiR‐590‐3p was identified as one of the highest confidence candidates (Figure 1a). In order to establish direct targeting between miR‐590‐3p and LRP6 mRNA 3′‐UTR, wild‐type (wtUTR) targeting sequence of miR‐590‐3p on 3′‐UTR of LRP6 mRNA and the mutated version (mutUTR) were cloned at the downstream of the luciferase open reading frame (ORF; Figure 1b). Luciferase constructs were transfected into HTR‐8/SVneo cells stably expressing miR‐590‐3p mimic (Figure 1c), followed by luciferase activity assay. We observed that the activity of wtUTR construct was greatly reduced in cells overexpressing miR‐590‐3p, whereas activity of mutUTR construct was unaltered under the same condition (Figure 1d).

**Figure 2:**
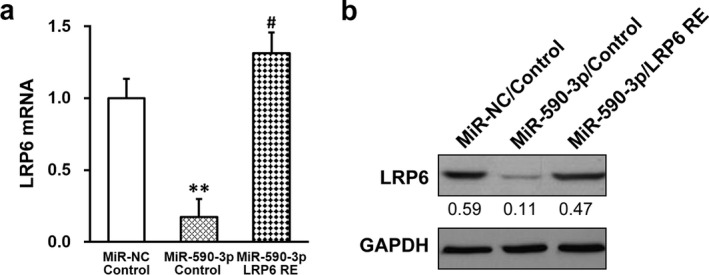
MiR‐590‐3p inhibits lipoprotein receptor‐related protein 6 (LRP6) expression via its 3′‐untranslated region (UTR). HTR‐8/SVneo cells were transduced with negative control miR (miR‐NC) or miR‐590‐3p mimic, respectively, following expression of empty plasmid (control) or plasmid expressing LRP6 independent of its 3′‐UTR (LRP6 RE). (a) mRNA and (b) protein levels of LRP6 were then subjected to RT‐PCR or Western blot analyses, respectively. Representative Western blot image was shown, with average densitometry analysis with relative to GAPDH band. Values are mean + *SD* from three independent experiments. **p < 0.01, compared to both miR‐NC/control and miR‐590‐3p/LRP RE. ^#^ *p* < 0.05, compared to miR‐NC/control

Next, expression of LRP6 was re‐introduced into HTR‐8/SVneo cells stably expressing miR‐590‐3p mimic, using a construct containing LRP6 ORF independent of its 3′‐UTR. Expression of this LRP6 RE construct readily restored both the mRNA and protein levels of LRP6 to its original levels in the absence of miR‐590‐3p mimic (Figure 2a,b). Taken results in Figures 1 and 2 together, we concluded that in HTR‐8/SVneo cells, miR‐590‐3p functioned to inhibit LRP6 expression via its 3′‐UTR. On the contrary, inhibition of endogenous miR‐590‐5p resulted a significant but slight decrease in its expression (Supporting Information Figure S1a), which nevertheless did not cause any noticeable difference on LRP6 expression (Supporting Information Figure S1b), likely because the endogenous expression levels of miR‐590‐3p in HTR‐8/SVneo and TEV1 cells were very low to start with (data not shown).

### 3.2 MiR‐590‐3p inhibits proliferation and induces apoptosis in HTR‐8/SVneo cells by repressing LRP6 expression

**Figure 3:**
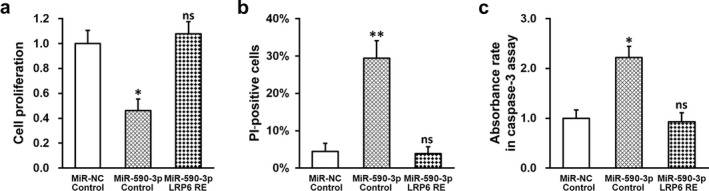
MiR‐590‐3p inhibits proliferation and induces apoptosis in HTR‐8/SVneo cells by repressing lipoprotein receptor‐related protein 6 (LRP6) expression. HTR‐8/SVneo cells were transduced with negative control miR (miR‐NC) or miR‐590‐3p mimic, respectively, following expression of empty plasmid (control) or plasmid expressing LRP6 independent of its 3′‐untranslated region (LRP6 RE). (a) Proliferation, (b) percentage of PI‐positive cells, and (c) relative absorbance rate of HTR‐8/SVneo cells were analyzed, respectively. Values are mean + *SD* from three independent experiments. **p < 0.01, *p < 0.05, compared to both miR‐NC/control and miR‐590‐3p/LRP RE. ns not significant, compared to miR‐NC/control

We subsequently examined the effect of miR‐590‐3p expression on the growth of HTR‐8/SVneo cells and discovered that proliferation of HTR‐8/SVneo cells with stable miR‐590‐3p expression was significantly inhibited compared to negative control miR (Figure 3a). Noteworthily, re‐introduction of LRP6 in miR‐590‐3p‐expressing HTR‐8/SVneo cells fully restored the proliferative capacity to its original levels. Next, apoptosis was assessed using Hoechst/PI staining and caspase‐3 colorimetric assay. MiR‐590‐3p expression in HTR‐8/SVneo cells resulted in significantly higher number of PI‐positive cells, indicating elevated apoptosis level (Figure 3b), which was then reduced back to control levels by LRP6 re‐expression. As expected, caspase‐3 colorimetric assay also indicated that the ratio of relative absorbance was significantly higher in HTR‐8/SVneo cells with miR‐590‐3p, which was again returned to comparable levels as the control (Figure 3c), suggesting higher apoptotic rate following miR‐590‐3p expression in HTR‐8/SVneo cells was also mediated by LRP6.

### 3.3 MiR‐590‐3p inhibits migration, invasion, and tube formation of HTR‐8/SVneo cells by repressing LRP6 expression

**Figure 4:**
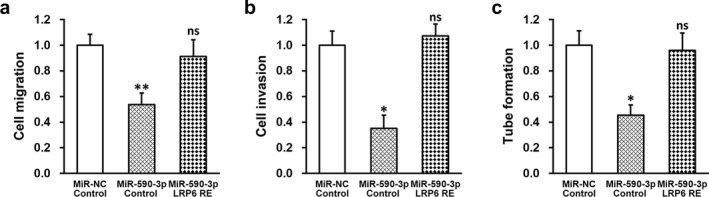
MiR‐590‐3p inhibits migration, invasion, and tube formation of HTR‐8/SVneo cells by repressing lipoprotein receptor‐related protein 6 (LRP6) expression. HTR‐8/SVneo cells were transduced with negative control miR (miR‐NC) or miR‐590‐3p mimic, respectively, following expression of empty plasmid (control) or plasmid expressing LRP6 independent of its 3′‐untranslated region (LRP6 RE). (a) Migration, (b) invasion, and (c) tube formation capacities of HTR‐8/SVneo cells were analyzed by wound healing, Matrigel invasion, and tube formation assays, respectively. Values are mean + *SD* from three independent experiments. **p < 0.01, *p < 0.05, compared to both miR‐NC/control and miR‐590‐3p/LRP RE. ns not significant, compared to miR‐NC/control

At this point, we have established the role of both miR‐590‐3p and LRP6 involved in the growth of HTR‐8/SVneo cells. We next investigated the effect of miR‐590‐3p and LRP6 in the migration, invasion, and tube formation capacities. To this end, HTR‐8/SVneo cells were stably transduced with negative control miR (miR‐NC) or miR‐590‐3p mimic, respectively, following expression of empty plasmid (control) or plasmid expressing LRP6 independent of its 3′‐UTR (LRP6 RE). Wound healing, Matrigel invasion, and tube formation assays were then respectively performed on the above cells (Figure 4a–c). MiR‐590‐3p significantly inhibited migration, invasion, and tube formation of HTR‐8/SVneo cells, and re‐introducing LRP6 in these cells markedly restored all capacities back to normal levels, demonstrating that miR‐590‐3p‐induced inhibition of migration, invasion, and tube formation was indeed mediated via repressing LRP6 expression. To further verify the role of miR‐590‐3p/LRP6 axis on trophoblast cell function, we repeated the above assays using another human first trimester EVT cell line TEV1. As expected, miR‐590‐3p significantly inhibited migration (Supporting Information Figure S2a), invasion (Supporting Information Figure S2b), and tube formation (Supporting Information Figure S2c) of TEV1 cells, and re‐introducing LRP6 also rescued all capacities back to normal levels.

### 3.4 MiR‐590‐3p downregulates expressions of MMPS and angiogenic factors in HTR‐8/SVneo cells via repressing LRP6

**Figure 5:**
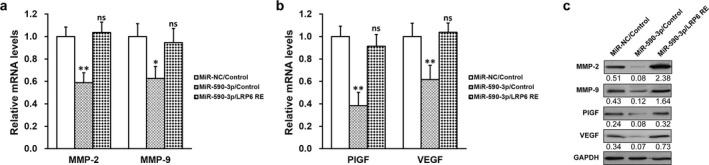
MiR‐590‐3p downregulates expressions of matrix metalloproteinases (MMPs) and angiogenic factors in HTR‐8/SVneo cells via repressing lipoprotein receptor‐related protein 6 (LRP6). HTR‐8/SVneo cells were transduced with negative control miR (miR‐NC) or miR‐590‐3p mimic, respectively, following expression of empty plasmid (control) or plasmid expressing LRP6 independent of its 3′‐untranslated region (LRP6 RE). (a) mRNA levels of MMP‐2, MMP‐9, (b) mRNA levels of angiogenic factors PlGF and VEGF, and (c) their protein levels were analyzed by RT‐PCR and Western blot, respectively. Representative Western blot image was shown, with average densitometry analysis with relative to GAPDH band. Values are mean + *SD *from three independent experiments. **p < 0.01, *p < 0.05, compared to both miR‐NC/control and miR‐590‐3p/LRP RE. ns not significant, compared to miR‐NC/control

Matrix metalloproteinase‐2 and MMP‐9 were reported to exert essential functions in the migration and invasion of trophoblast cells. We therefore examined the mRNA levels of MMP‐2 and MMP‐9 in HTR‐8/SVneo cells, and observed that both of them were significantly downregulated by miR‐590‐3p, and rescued by LRP6 re‐expression (Figure 5a). In addition, as angiogenesis of trophoblasts is closely associated with various factors such as VEGF and PlGF, we also assess their mRNA levels. As expected, miR‐590‐3p in HTR‐8/SVneo cells markedly decreased the mRNA levels of PlGF and VEGF, which were also restored by LRP6 expression (Figure 5b). Similarly, miR‐590‐3p in TEV1 cells also significantly repressed the mRNA levels of both MMP‐2 and MMP‐9 (Supporting Information Figure S3a), as well as angiogenic factors PlGF and VEGF (Supporting Information Figure S3b), all of which could be almost fully rescued by LRP6 expression. We also noticed that protein expressions of all the above factors followed the same trend of their mRNAs (Figure 5c).

### 3.5 MiR‐590‐3p inhibits Wnt pathway factor β‐catenin in HTR‐8/SVneo cells via repressing LRP6

**Figure 6:**
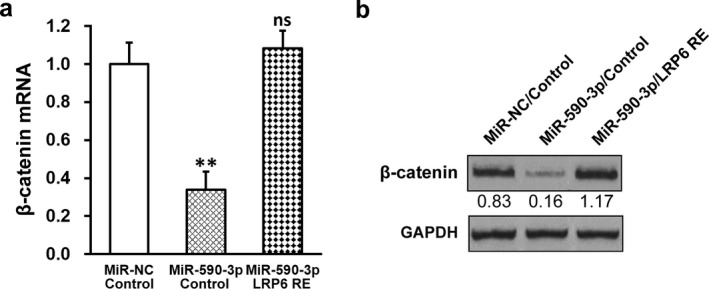
MiR‐590‐3p inhibits Wnt pathway factor β‐catenin in HTR‐8/SVneo cells via repressing lipoprotein receptor‐related protein 6 (LRP6). HTR‐8/SVneo cells were transduced with negative control miR (miR‐NC) or miR‐590‐3p mimic, respectively, following expression of empty plasmid (control) or plasmid expressing LRP6 independent of its 3′‐untranslated region (LRP6 RE). (a) mRNA and (b) protein levels of β‐catenin were analyzed by RT‐PCR and Western blot, respectively. Representative Western blot image was shown, with average densitometry analysis with relative to GAPDH band. Values are mean + *SD* from three independent experiments. **p < 0.01, compared to both miR‐NC/control and miR‐590‐3p/LRP RE. ns not significant, compared to miR‐NC/control

Besides affecting MMPs and angiogenic factors, LRP6 is also involved in the Wnt/β‐catenin signaling during vascular development; hence, we next examined the expression of β‐catenin, an essential factor of the Wnt signaling pathway, in HTR‐8/SVneo cells. We found that β‐catenin mRNA and protein levels were significantly reduced by miR‐590‐3p (Figure 6a,b). Again, as expected, LRP6 re‐expression fully restored β‐catenin expression to its original levels (Figure 6a,b and Supporting Information Figure S4). The above results clearly suggested that miR‐590‐3p downregulated expressions of MMPs, angiogenic factors, and Wnt pathway factor β‐catenin in HTR‐8/SVneo cells via repressing LRP6.

## 4 DISCUSSION

Invasive behavior of trophoblasts is thought to contribute to correct maternal spiral artery remodeling. Increasing evidences have suggested that defective modification of maternal vascular development as a result of shallow trophoblast invasion is correlated with to obstetric complications (Kaufmann et al., 2003; van den Brule et al., 2005). Tsatas, Baker, and Rice, (1997 have found that in human term gestational tissues, LRP expression was confined to chorion trophoblasts, as well as those in the villous and extravillous tissues, suggesting potential tissue‐specific function of LRP. In our own study, we observed a dramatic decrease in the invasive and migrative capacities, along with changes in proliferation and apoptosis of trophoblast cells, after downregulation of LRP6 expression by miR‐590‐3p in HTR‐8/SVneo cells. These findings are consistent with studies in various tumor models (Fan, Shi, Li, & Kuang, 2017; Jackson et al., 2016; Ma, Lu, Chen, Xu, & Li, 2017). For instance, antibody against LRP6 was able to inhibit tumor growth (Jackson et al., 2016). In triple negative breast cancer, LRP6 was able promote tumor migration and invasion by altering the expression and function of S100A4 (Ma et al., 2017). Inhibition of LRP6 by miRNA‐454 in pancreatic ductal adenocarcinoma exerted antiangiogenic and antimetastatic effects (Fan et al., 2017). Invasion of trophoblasts during early pregnancy requires MMP‐2 and MMP‐9 (Staun‐Ram, Goldman, Gabarin, & Shalev, 2004). Our data demonstrated that LRP6 inhibition by miR‐590‐3p suppressed protein expressions of both MMP‐2 and MMP‐9, in addition to their mRNA levels, demonstrating that the major effect of LRP6 is likely to be realized by promoting activities of these two enzymes.

Replacing the endovascular trophoblasts is considered a critical process during remodeling of spiral artery (Whitley & Cartwright, 2010). Following invasion into the maternal deciduas, EVTs differentiate into endovascular trophoblasts and convert spiral arteries to vessels with lower resistance during pregnancy. Our own study revealed a defective tube formation of HTR‐8/SVneo cells after miR‐590‐3p‐induced LRP6 repression in vitro. Therefore, insufficient maternal spiral artery conversion, due to LRP6 inhibition, may contribute to impaired placentation. In addition, reductions in angiogenic factor expression during the first trimester have been involved in the manifestation of placental vascular complications during later term (Farina et al., 2008). The downregulation of both angiogenic factors, PlGF and VEGF, is involved in the pathogenesis of preeclampsia (Farina et al., 2008). We therefore further examined expressions of both PlGF and VEGF, and found inhibition of PlGF transcription following miR‐590‐3p‐induced LRP6 suppression. Our data demonstrated that expression of miR‐590‐3p was negatively, whereas LRP6 was positively correlated with those of PlGF and VEGF, suggesting that miR‐590‐3p and LRP6 likely exhibit functions as anti‐ and pro‐angiogenic factors, respectively, during the remodeling of spiral artery remodeling. Investigations are presently on the way to decipher the regulatory pathways linking LRP6 with PlGF and VEGF.

As for the potential molecular mechanisms, we speculate that LRP6 may affect trophoblast functions in spiral artery remodeling via the Wnt/β‐catenin signaling pathway, which is involved in angiogenesis and vascular development as reported (Knofler & Pollheimer, 2013). In Xenopus embryos, dominant‐negative LRP6 could inhibit signal transduction of several Wnt proteins, whereas on the other hand, overexpression of LRP6 synergized with Wnt to stimulate Wnt/β‐catenin signaling (Tamai et al., 2000). Early placental development is correlated with rapid generation of various subtypes of trophoblasts to form distinct functioning villi (Georgiades, Ferguson‐Smith, & Burton, 2002; Red‐Horse et al., 2004). Adaption of the maternal uterus to pregnancy requires extensive tissue remodeling, such as stromal cells differentiation, immunological modifications, and angiogenesis. These critical events occur in the secretary phase of the menstrual cycle, throughout implantation and during early placental development. Given that Wnt signaling plays an essential role in tissue homeostasis and organ development, it is not surprising that Wnt signaling exerts important functions in the formation, differentiation, and growth of uterus. Gene targeting study has demonstrated that β‐catenin is required for uterine development (Arango et al., 2005). For instance, studies using mouse models have demonstrated that Wnt signaling is a key player in the activation and implantation of blastocysts (Mohamed et al., 2005). In vitro studies using trophoblasts have also provided data indicating the involvement of Wnt signaling in adhesion, invasion, and differentiation of trophoblasts. In addition, experiments in T‐cell factor expression in human placenta demonstrated that the Wnt pathway is involved in the invasive differentiation of trophoblasts (Pollheimer et al., 2006). Moreover, treatment with a Wnt activator potentiated the invasion of cytotrophoblasts and trophoblasts, and stimulated their migration in villous explant culture, which could be inhibited by Dkk1, a Wnt signaling inhibitor (Pollheimer et al., 2006; Sonderegger et al., 2010). Our current study showed that inhibited Wnt signaling correlates with repressed invasion and migration of HTR‐8/SVneo cells after LRP6 downregulation by miR‐590‐3p and further validated that LRP6 is critically involved in trophoblast‐dependent maternal spiral artery remodeling, which is potentially mediated by Wnt/β‐catenin signaling pathway. Experiments to reveal other downstream effectors of LRP6 in the Wnt/β‐catenin signaling pathway are currently underway, in order to provide a comprehensive understanding on the mechanisms linking LRP6 with Wnt/β‐catenin during maternal spiral artery remodeling.

Although miR‐590‐3p has been reported to be involved in various other human diseases, it is a novel miR that is only implicated in trophoblast‐dependent maternal spiral artery for the first time. Several important genes were found to be the direct target of miR‐590‐3p in different disease models. For instance, in cancers, miR‐590‐3p suppresses hepatocellular carcinoma growth by targeting TEAD1 (Ge & Gong, 2017), inhibits epithelial–mesenchymal transition in intrahepatic cholangiocarcinoma by targeting SIP1 (Zu et al., 2017), and represses migration, invasion, and epithelial–mesenchymal transition in glioblastoma multiforme by targeting ZEB1 and ZEB2 (Pang, Zheng, Zhao, Xiu, & Wang, 2015). In myocarditis, miR‐590‐3p was also identified as a novel miRNA that could target nuclear factor κ‐B in vivo (Zhao et al., 2015). On a related note, in endothelial progenitor cells, miR‐590‐3p likely inhibited interleukin‐18 expression to promote angiogenesis in vivo (Li et al., 2017). In our current in vitro cell culture model of using trophoblast cell line HTR‐8/SVneo, miR‐590‐3p was demonstrated to directly target LRP6 to downregulate its expression. This new discovery serves yet as a new aspect on the multifacade functions of miR‐590‐3p and is also consistent with the biological principle of mRNA targeting by miRNAs.

In summary, we evaluated the functions of LRP6 and miR‐590‐3p in a trophoblast cell line HTR‐8/SVneo in vitro and observed miR‐590‐3p could significantly impair migration, invasion, and tube formation by directly downregulating LRP6 expression, which was likely mediated by downstream effectors including MMPs and angiogenic factors PlGF and VEGF. Further, we discovered that miR‐590‐3p‐induced LRP6 inhibition suppressed the Wnt signaling pathway. Our findings, for the first time, demonstrated the function of LRP6 and identified the critical role of a novel miR‐590‐3p in vitro, hinting its potential implication in invasion of trophoblasts and endovascular formation. Our data suggest that LRP6 and miR‐590‐3p may act in opposing roles to regulate the trophoblast‐dependent remodeling of maternal spiral artery.

## CONFLICTS OF INTEREST

The authors declare that they have no conflict of interest.
