# Supplementary material for: MicroRNA‐590‐3p inhibits trophoblast‐dependent maternal spiral artery remodeling by repressing low‐density lipoprotein receptor‐related protein 6
Source: Mol Genet Genomic Med. 2018 Nov 8;6(6):1124–33. doi: 10.1002/mgg3.491 (PMC6305632; doi:10.1002/mgg3.491)
Supplement: Supplementary file 1 [file MGG3-6-1124-s001.docx]

**Supplementary Materials**





**Figure S1. MiR-590-3p inhibition exerts insignificant effect.**

HTR-8/SVneo cells were transduced with control or miR-590-3p inhibitor (anti-miR-590), respectively, followed by assessments of (A) endogenous miR-590-3p and (B) LRP6 mRNA expression levels. Values are mean+SD from three independent experiments. * p<0.05, ns not significant, compared to control.





**Figure S2. MiR-590-3p inhibits migration, invasion and tube formation of TEV1 cells by repressing LRP6 expression.**

Human first trimester EVT cell line TEV1 cells were transduced with negative control miR (miR-NC) or miR-590-3p mimic, respectively, following expression of empty plasmid (control) or plasmid expressing LRP6 independent of its 3’-UTR (LRP6 RE). (A) Migration, (B) invasion and (C) tube formation capacities of TEV1 cells were analyzed by wound healing, Matrigel invasion and tube formation assays, respectively. Values are mean+SD from three independent experiments. ** p<0.01, * p<0.05, compared to both miR-NC/control and miR-590-3p/LRP RE. ns not significant, compared to miR-NC/control.





**Figure S3. MiR-590-3p down-regulates expressions of MMPs and angiogenic factors in TEV1 cells via repressing LRP6.**

Human first trimester EVT cell line TEV1 cells were transduced with negative control miR (miR-NC) or miR-590-3p mimic, respectively, following expression of empty plasmid (control) or plasmid expressing LRP6 independent of its 3’-UTR (LRP6 RE). (A) mRNA levels of MMP-2, MMP-9, (B) mRNA levels of angiogenic factors PlGF and VEGF were analyzed by RT-PCR. Values are mean+SD from three independent experiments. ** p<0.01, * p<0.05, compared to both miR-NC/control and miR-590-3p/LRP RE. ns not significant, compared to miR-NC/control.


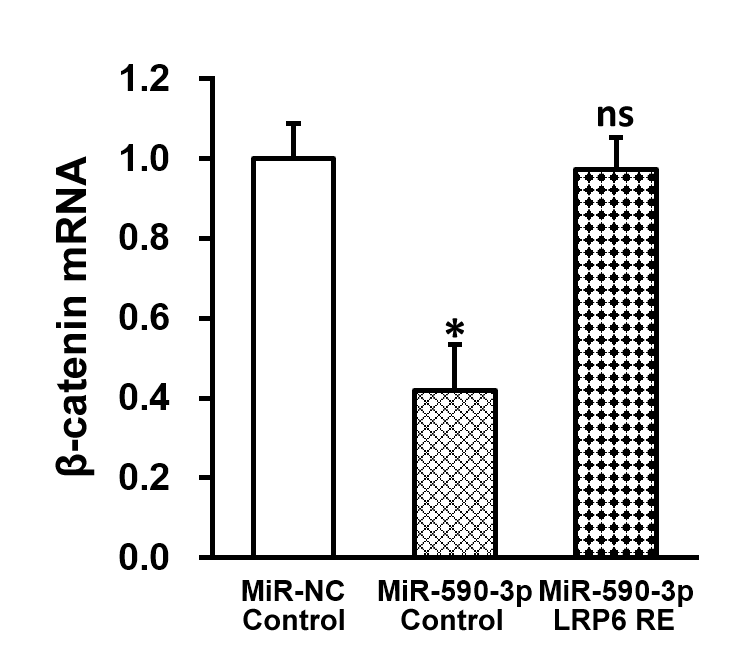


**Figure S4. MiR-590-3p inhibits Wnt pathway factor β-catenin in TEV1 cells via repressing LRP6.**

Human first trimester EVT cell line TEV1 cells were transduced with negative control miR (miR-NC) or miR-590-3p mimic, respectively, following expression of empty plasmid (control) or plasmid expressing LRP6 independent of its 3’-UTR (LRP6 RE). mRNA level of β-catenin was analyzed by RT-PCR. Values are mean+SD from three independent experiments. * p<0.05, compared to both miR-NC/control and miR-590-3p/LRP RE. ns not significant, compared to miR-NC/control.
